# Sow performance in multi-suckling pens with different management routines

**DOI:** 10.1186/s13028-018-0364-x

**Published:** 2018-02-08

**Authors:** Ola Thomsson, Ulf Magnusson, Ann-Sofi Bergqvist, Lena Eliasson-Selling, Ylva Cecilia Björnsdotter Sjunnesson

**Affiliations:** 1 Division of Reproduction, Department of Clinical Sciences, Swedish University of Agricultural Sciences, SLU, P.O. Box 7054, 750 07 Uppsala, Sweden; 2 Present Address: The Swedish Research Council for Environment, Agricultural Sciences and Spatial Planning, P.O. Box 1206, 111 82 Stockholm, Sweden; 3 Centre for Reproductive Biology in Uppsala, CRU, P.O. Box 7054, 750 07 Uppsala, Sweden; 4 Farm & Animal Health, Kungsängens Gård, 753 23 Uppsala, Sweden

**Keywords:** Back fat, Estrus, Group housing, Lactational estrus, Litter size, Pig reproduction, Weaning to estrus interval

## Abstract

Production systems with group housing of sows during a part of the lactation are used in certified organic production and can increase the occurrence of lactational estrus thus making batch-wise breeding difficult. The aim of this study was to investigate the occurrence of lactational estrus and time at return to estrus after weaning by following the performance of the sow (change in body weight, back fat and litter size) in three different management routines. The sows and their litters were moved from individual to multi-suckling pen at one (W1; n = 14), two (W2; n = 13), or 3 weeks (W3; n = 16) post farrowing. All sows had a total lactation of 6 weeks. Ovulation was monitored by analysis of fecal progesterone metabolites. Only one sow (W3) ovulated during lactation. Sows in the W2 and W3 groups had a shorter weaning-to-standing estrus interval than W1-sows (2.6 ± 0.3; 2.7 ± 0.2 and 4.0 ± 0.3 days respectively, P < 0.001). The W1-sows and piglets might have kept their nursing bond more intact all through the group housing since the piglets were completely dependent on the nursing at the time of their move to the group pen, thereby staying in lactational anestrus and retaining standard weaning-estrous interval. There was no difference in litter size at grouping or at weaning between management routines and parities. Third and later parity sows had significantly thicker back fat at farrowing and at weaning than 1st and 2nd parity sows (P < 0.05). In conclusion, the occurrence of lactational estrus can be low in a multi-suckling pen and the interval between farrowing and move to a multi-suckling pen can affect the weaning to estrus interval. The short weaning-to-standing estrus interval seen in W2 and W3 suggests that estrus detection should start immediately post weaning for sows kept in multi-suckling pens.

## Findings

Production systems with group housing of sows during a part of the lactation are used in some niche production systems such as certified organic production [1]. Keeping lactating sows in multi-suckling pens rather than individual farrowing pens can impair production through increased occurrence of lactational estrus [2, 3]. The return to estrus usually occurs in late lactation, particularly among older sows and sows in good body condition, and this late occurrence results in a prolonged weaning-to-service interval, making batch-wise breeding difficult [2, 4, 5].

The aims of this study were to investigate (1) occurrence of estrus during lactation and weaning-to-estrus interval in three management routines and (2) differences in other aspects of sow performance, i.e. weight, back fat, weight loss, and back fat loss during lactation, and litter size at onset of group housing and at weaning.

The study was performed at Funbo Lövsta, Uppsala. A total of 43 Yorkshire sows were included in the study. The sow and piglets spent either 1 (W1), 2 (W2), or 3 (W3) weeks in an individual farrowing pen (available free space for the sow was 6.4 m^2^ and straw bedding was provided on top of hard floor surface) before being group housed in a multi-suckling pen. Each management routine was repeated once. Due to the variation in farrowing dates, it took between 1 and 3 days for all sows in each management routine to reach the multi-suckling pen. The time of the move for individual sows was included in the statistical information as “subset”. Weaning occurred at 6 weeks post farrowing. Descriptions of the sows at farrowing and onset of group housing can be found in Tables 1 and 2.

**Table 1:** Differences between management routines (W1–W3) with regard to litter size, lactation length, weight, and back fat at farrowing and weaning (Lsmeans ± S.E), and parity (number of sows)

	W1	W2	W3	P
Litter size				
Live born	12.5 ± 0.7	13.3 ± 0.8	11.8 ± 0.7	n.s.
At grouping	10.4 ± 0.6	10.4 ± 0.7	9.8 ± 0.6	n.s.
At weaning	9.0 ± 0.6	9.6 ± 0.6	9.6 ± 0.5	n.s.
Lactation period (days)	44.2 ± 0.6	43.6 ± 0.6	43.7 ± 0.6	n.s.
Sow weight (kg)				
At farrowing	257.7 ± 7.8	266.7 ± 8.2	256.1 ± 7.6	n.s.
At weaning	246.8 ± 7.3^ab^	263.6 ± 7.6^a^	233.5 ± 7.1^b^	< 0.05
Difference	− 11.2 ± 5.7^ab^	− 1.0 ± 6.1^a^	− 22.4 ± 5.5^b^	< 0.05
Back fat (mm)				
At farrowing	17.5 ± 1.2	17.4 ± 1.3	19.0 ± 1.2	n.s.
At weaning	14.0 ± 1.0	16.8 ± 1.1	15.2 ± 1.0	n.s.
Difference	− 3.6 ± 0.8^a^	− 0.5 ± 0.9^b^	− 3.8 ± 0.8^a^	< 0.05
1st parity	3	2	6	n.s.
2nd parity	6	4	4	n.s.
> 2nd parity	5	7	6	n.s.

Different superscripts within rows indicate significant differences

*n.s.* non-significant (P > 0.5)

W1 = The sows and their litters were moved from individual to multi-suckling pen at 1 week post farrowing, W2 = The sows and their litters were moved from individual to multi-suckling pen at 2 weeks post farrowing, W3 = The sows and their litters were moved from individual to multi-suckling pen at 3 weeks post farrowing

**Table 2:** Differences between parities with regard to litter size, lactation length, weight, and back fat at farrowing and weaning (Lsmeans ± S.E)

	1st party sows	2nd parity sows	> 2nd parity sows	P
Litter size				
Live born	12.4 ± 0.9	12.6 ± 0.7	12.6 ± 0.7	n.s.
At grouping	10.5 ± 0.7	10.1 ± 0.6	10.0 ± 0.6	n.s.
At weaning	9.3 ± 0.7	10.0 ± 0.6	9.0 ± 0.5	n.s.
Lactation period (days)	43.5 ± 0.4	43.9 ± 0.4	44.1 ± 0.4	n.s.
Sow weight (kg)				
At farrowing	213.3 ± 7.2^a^	264.4 ± 6.1^b^	302.9 ± 6.3^c^	< 0.001
At weaning	210.9 ± 8.1^a^	244.3 ± 6.5^b^	288.7 ± 6.5^c^	< 0.001
Difference	− 0.2 ± 6.3^a^	− 20.1 ± 5.0^b^	− 14.4 ± 5.1^ab^	< 0.05
Back fat (mm)				
At farrowing	16.1 ± 1.1^a^	18.0 ± 1.0^a^	20.3 ± 1.0^b^	< 0.05
At weaning	12.6 ± 1.3^a^	14.8 ± 1.0^a^	18.5 ± 1.0^b^	< 0.05
Difference	− 3.0 ± 1.0	− 3.0 ± 0.8	− 1.8 ± 0.8	n.s.

Different superscripts within rows indicate significant differences

*n.s.* non-significant (P > 0.05)

**Fig. 1:**
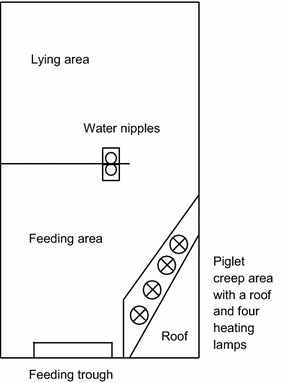
Illustration of the multi-suckling pen (reproduced with the permission of BioMed Central original publisher)

Three multi-suckling pens were constructed in an uninsulated barn as described previously [6]. In brief, space allowance per sow in the multi-suckling pen was according to standards for Swedish organic breeding [1] and ranged from 7.8 to 12.5 m^2^ depending on the number of sows per pen (5–8 sows/pen). The design is illustrated in Fig. 1. The bedding consisted of straw in the lying area and peat in the feeding area. During the entire lactation period, the sows were fed ad libitum with dry feed in a feeding trough that was accessible to the piglets as well. In addition, sows and piglets had access to hay at all times. Water was provided ad libitum.

During the group housing period daily manual estrus detection tests were performed by inspection vulva swelling and reddening and by applying back pressure. Post weaning, manual estrus detection was performed twice a day with a boar present until estrus was detected.

Fecal samples were collected from the rectum every third day from day 21 post farrowing until the day of weaning and stored at − 20 °C until analysis.

Sows were slaughtered after the first reported standing estrus post weaning and the reproductive tract was retrieved and examined macroscopically.

At farrowing and weaning, each sow’s back fat thickness was measured ultrasonically by experienced staff using a Krautkramer USM22 device (GE Inspection Technology GmBH, Hürth, Germany) at the last rib, between 7 and 8 cm from the middle of the back [7].

Most of the progesterone in feces is present in a metabolized form [8] and progesterone and its metabolites are referred to hereafter as progestin. The method used for extracting progestin from feces was modified from Palme et al. [9] and Wasser et al. [10]. One gram feces was mixed with 10 mL methanol (≥ 99.9%) and the slurry obtained was shaken for 1 h, followed by 15 min of centrifugation at 3000×*g* at room temperature. Three milliliters of the supernatant was then stored at − 20 °C until analysis. The analysis was conducted using a solid-phase 125I-radioimmunassay (Coat-a-count Progesterone, Siemens Healthcare Diagnostic Inc, Los Angeles, CA, USA) previously validated for pig feces [7]. The analysis was performed according to the manufacturer’s instructions. The inter-assay coefficient of variation for high, medium, and low concentrations was 12, 12 and 14%, respectively. The intra-assay coefficient of variation was below 10%, within the working range of 0.3–127 nmol/L.

All statistical analyses were performed using SAS software ver. 9.3 (SAS Inst., Cary, NC, USA). To analyze differences between management routines regarding days from weaning to standing estrus, the procedure Mixed was used. Fixed effects included were management routine, repeat, parity, and subset. Litter size at group housing was added as covariate. Random effect was repeat nested within management routine and subset. Subset did not influence the outcomes. To analyze progestin profiles, the 99th and 97.5th ‰ were calculated from the progestin concentration in every fecal sample [7] collected at day 21 post farrowing. The progestin concentration in the fecal samples collected at day 21 post farrowing was considered to represent anestral levels of progesterone. The 99th ‰ was 67 nmol/L and the 97.5th ‰ was 48 nmol/L. Ovulation was considered to be possible if one fecal sample had a concentration above 67 nmol/L or if two consecutive fecal samples had a concentration above 48 nmol/L. P < 0.05 were considered significant.

**Fig. 2:**
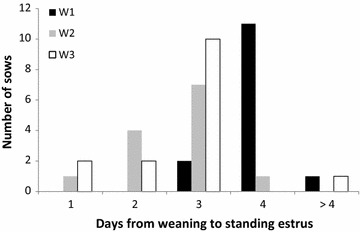
Days from weaning to standing estrus distribution in different management routines (W1–W3). The interval from weaning to standing estrus was shorter for W2 (2.7 days ± 0.2; P < 0.05) and W3 (2.4 days ± 0.2; P < 0.001) than W1 (3.8 days ± 0.2). There was no difference between W2 and W3

Number of days from weaning to standing estrus differed between the three management routines as illustrated in Fig. 2. The weaning-to-standing estrus interval of sows in management routine W2 (2.6 days ± 0.3; P < 0.001) and W3 (2.7 days ± 0.2; P < 0.001) was shorter than that of W1-sows (4.0 days ± 0.3; P < 0.001). The majority of weaned sows usually display standing estrus on days four and five post weaning [11–14]. The shorter weaning-to-standing estrus interval for management routines W2 and W3 could therefore be of practical importance. A short weaning-to-standing estrus interval indicates that estrus detection needs to start immediately post weaning since failure to detect the start of standing estrus or the entire standing estrus display can have a negative effect on overall pig production economics. The W1 sows and piglets might have kept their nursing bond more intact all through the group housing since the piglets were completely dependent on the suckling at the time of their move to the group pen, thereby maintaining the lactational anestrus and retaining standard weaning-estrous interval.

**Fig. 3:**
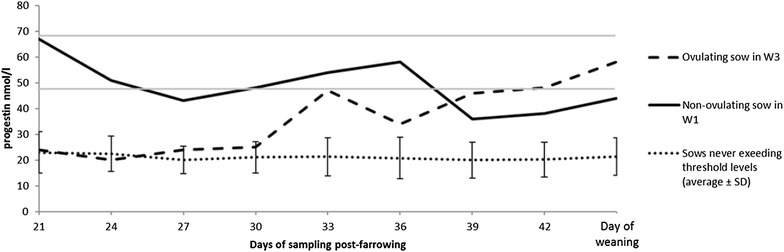
Fecal progestin concentration profile. Samples from every third day with start on day 21 post-farrowing until the day of weaning for management routines W1, W2 and W3. Threshold levels are visualized (99th ‰ with 67 nmol/L and 97.5th with 48 nmol/L). Mean values of all sows that did not exceed threshold levels as well as values for two sows that had two samples or more that exceeded the lower threshold level are shown

Mean fecal progestin concentrations for all sows and for two individual sows, one in management routine W1 and one in the W3 routine, that had two samples or more that exceeded the lower threshold level are presented in Fig. 3. Only the W3-sow had a confirmed ovulation (corpora lutea from lactation visible in ovaries at slaughter) during lactation and no explanation was found for the increased progestin concentrations in the W1-sow. The ovulating sow was a 1st parity sow with 12 piglets at birth and eight piglets at weaning. The ovulating sow gained 14 kg and lost 1.5 mm back fat during lactation, resulting in a back fat depth of 18 mm at weaning. The weight gain, the back fat depth at weaning, and low back fat loss during lactation for the ovulating sow indicate that the sow had the potential to ovulate during lactation [2]. Since the sows were slaughtered as soon as possible after onset of standing oestrus after weaning, not all sows had ovulated when the reproductive tracts were examined. The mean number of ovulations/sow were 28, 25 and 31 and the mean uterus weight was 851, 785 and 851 g in W1, W2 and W3 respectively. None of the sows in any management routine displayed standing estrus during the lactation period. There is great variation in occurrence of lactational estrus among studies reported in the literature, ranging from 0% [15] to 100% when a boar is present [16, 17]. Another factor known to increase the occurrence of lactational estrus is a pen that allows many social interactions among sows and has many compartments, which reduces the interaction between the sow and her piglets [18]. Pen design might therefore have caused the sows to remain in lactational anestrus during the lactation period in the multi-suckling pen. Hultén et al. [3] investigated sows housed under organic conditions and found the lowest occurrence of lactational estrus during the fall which coincides with when this study was conducted. This might have contributed to the lack of lactational estrus seen in the present study. Regardless of the reason, this study suggests that lactational estrus does not have to be a major problem when sows are kept in multi-suckling pens.

There were no differences between management routines or between parities in the number of live born piglets and litter size at grouping and at weaning (Tables 1 and 2). Body condition loss during lactation also seemed influenced by timing of grouping (with the W2-sows having less body weight and back fat loss) but could be due to individual variation and that the W2 group had numerically less first parity sows than the W1 and W3 group (Table 1). The > 2nd parity sows were heavier and had more back fat than 1st and 2nd parity sows at farrowing and weaning (Table 2). This difference is not surprising, as young sows are still growing and have not accumulated as much back fat as older sows [19, 20]. There was no apparent connection between back fat loss or body weight loss and weaning-to-standing estrus interval. Previous studies have also reported a lack of significant correlation between back fat loss and weaning-to-standing estrus interval [19, 21].

In conclusion, sows group housed at 2 or 3 weeks post farrowing in multi-suckling pens had a shorter interval from weaning to standing estrus than sows group housed at 1 week post farrowing, which had a normal interval. This could be due to differences in nursing–suckling establishment and interaction in the group housing pen. These results suggest that estrus detection should start immediately post weaning for sows kept in multi-suckling pens, in order to avoid missing the onset of estrus. The occurrence of lactational estrus was found to be low in these multi-suckling pens.
